# Examining long-term repetition priming effects in spoken word recognition using computer mouse tracking

**DOI:** 10.3389/fpsyg.2022.1074784

**Published:** 2023-01-05

**Authors:** Samantha E. Tuft, Sara Incera, Conor T. MᶜLennan

**Affiliations:** ^1^Language Research Laboratory, Department of Psychology, Cleveland State University, Cleveland, OH, United States; ^2^Multilingual Laboratory, Department of Psychology, Eastern Kentucky University, Richmond, KY, United States

**Keywords:** priming, spoken word recognition, mouse tracking, long-term repetition, online research

## Abstract

Language researchers in a variety of disciplines have used priming as a tool to investigate theoretical questions. In spoken word recognition, long-term repetition priming effects have been obtained across a number of behavioral tasks (e.g., lexical decision, shadowing). Repeated – primed – words are responded to more efficiently than new – unprimed – words. However, to our knowledge, long-term repetition priming effects have not been examined using computer mouse tracking, which would provide data regarding the time course of long-term repetition priming effects. Consequently, we compared participants’ lexical decision responses using a computer mouse to primed and unprimed words. We predicted that participants would respond more efficiently to primed words compared to unprimed words. Indeed, across all of the dependent variables investigated (accuracy, reaction time, mouse trajectories) and across environments (in person, online), participants responded more efficiently to primed words than to unprimed words. We also performed additional exploratory analyses examining long-term repetition priming effects for nonwords. Across environments (in person, online), participants had more errors to primed nonwords than to unprimed nonwords, but there were no differences in reaction times and mouse trajectories. The current data demonstrating long-term repetition priming effects in mouse tracking are expected to motivate future investigations examining the time course of various long-term repetition priming effects for both words and nonwords.

## Introduction

Priming refers to the notion that a stimulus is responded to differently if it had been presented previously. According to the *APA Dictionary of Psychology* (VandenBos, 2007), priming refers to:

the effect in which recent experience of a stimulus facilitates or inhibits later processing of the same or a similar stimulus. In repetition priming, presentation of a particular sensory stimulus increases the likelihood that participants will identify the same or a similar stimulus later in the test. (p. 833)

The dictionary also explains that “the effects of repetition priming (e.g., changed speed of response, number of response errors) can occur without explicit memory of the first stimulus” (VandenBos, 2007, p. 906). Indeed, priming is considered a type of implicit or non-declarative memory.

Language researchers in a variety of areas have used priming as a tool to investigate effects of theoretical interest (including semantic priming, e.g., Holcomb and Neville, 1990; and syntactic priming, e.g., Pickering and Branigan, 1998), as have psychological scientists investigating other areas, such as memory (Ratcliff and McKoon, 1988). Priming has also been used in studies with clinical implications (Pagani et al., 2014). Slowiaczek and Pisoni (1986) were among the first to report repetition priming effects in spoken word recognition using an auditory lexical decision task. In an auditory lexical decision task, the participant must decide on each trial whether a stimulus is a real word or a nonword. Typically, participants are instructed to do so as quickly and accurately as possible. The logic underlying the task is that the efficiency with which participants respond reflects the relative ease of lexical access. Consequently, if higher frequency words are more readily accessible to participants, then words higher in frequency should be recognized – and responded to in a lexical decision task – more efficiently (faster, more accurately, or both) than lower frequency words. These effects are exactly what Slowiaczek and Pisoni – and numerous other researchers – have reported.

In spoken word recognition, listeners typically respond more efficiently (e.g., faster, more accurately, or both) to a recently heard word than to a new word. In short-term priming, repeated words could be separated by seconds or less. In the long-term repetition paradigm, listeners are presented with two separate blocks of spoken words, often with a break (which may include a filler task) between the two blocks. The difference between the two approaches is that in the long-term paradigm repeated words are separated by at least a couple of minutes, possibly much longer. Long-term repetition priming effects in spoken word recognition have been obtained across a number of behavioral tasks, including lexical decision and shadowing (see, e.g., MᶜLennan and Luce, 2005). Despite the widespread use of the long-term repetition priming paradigm by researchers interested in spoken word recognition (e.g., Church and Schacter, 1994; Luce and Lyons, 1998; MᶜLennan and Luce, 2005; MᶜLennan and González, 2012; Kessler and Moscovitch, 2013; Dufour and Nguyen, 2014; Maibauer et al., 2014; Tuft et al., 2018), the time course of long-term repetition priming effects is unknown. To our knowledge, there is no published empirical investigation aimed at understanding how the advantage for primed items (e.g., words), relative to unprimed items, evolves over time using the long-term repetition priming paradigm and computer mouse tracking. However, in a 2018 investigation of short-term semantic priming, Kang et al. (2018) demonstrated that tracking hand/computer mouse movements during a lexical decision task can “shed light on cognitive processes as they unfold in real time” (p. 1506).

Mouse tracking allows researchers to measure how a response unfolds continuously throughout a trial. As Freeman (2018) reported, “mouse tracking has become a popular method across psychological science” (p. 315). Mouse tracking studies have been conducted to investigate a wide range of topics like stereotypes (Hehman et al., 2014), food choices (Stillman et al., 2017), bilingual language processing (Incera and MᶜLennan, 2018b), and the bilingual advantage in cognitive processing (Incera and MᶜLennan, 2016, 2018a). Spivey et al. (2005) were the first to use this technique when investigating spoken word recognition. In their study, participants moved the mouse toward the word “candy” when hearing the spoken word “candle” (one example), providing support for models of spoken word recognition in which there is continuous attraction toward similar sounding words (phonological neighbors).

Barca and Pezzulo (2012) were the first to use mouse tracking to examine participants’ performance in a visual lexical decision task. These researchers found that participants’ mouse movements in response to low frequency words moved closer to the nonword response option than participants’ mouse movements in response to high frequency words. Soon after this initial study, Krestar et al. (2013) were the first to use mouse tracking to examine listeners’ performance in an auditory lexical decision task. Krestar and colleagues found that listeners’ mouse movements were more efficient for words relative to nonwords. Both studies demonstrate the value in using mouse tracking in order to learn about the real-time processing dynamics throughout the course of word recognition.

In the current study, we combined the long-term repetition priming paradigm with mouse tracking and the lexical decision task in order to provide new insights into how listeners recognize spoken words. Our study, including our hypotheses and planned analyses, was preregistered on the Open Science Framework, hosted by the Center for Open Science. This initial investigation is expected to motivate future investigations examining the time course of long-term repetition priming using computer mouse tracking.

### Hypotheses

Participants’ lexical decision responses to primed words are expected to be more efficient than responses to unprimed words in one or more of the following dependent variables:

Accuracy: Fewer incorrect responses are expected in the primed word condition than in the unprimed word condition.

Reaction Time: Faster responses are expected in the primed word condition than in the unprimed word condition.

Mouse Movement Trajectories: Intercept, slope differences, or both are expected, such that the primed word condition is more efficient (higher intercept, steeper slope, or both) than the unprimed word condition.

## Experiment 1

### Method

#### Participants

A total of 46 undergraduate participants^1^ (31 women, *M*_Age_  = 19.61, *SE*_Age_ = 0.31) from Cleveland State University were recruited in exchange for research participation credit. All participants were right-handed, native speakers of American English, with no reported hearing or speech disorders. All participants provided their written informed consent before participation and the procedures were approved by the Institutional Review Board at Cleveland State University.

#### Stimuli

The stimuli consisted of 80 word and 80 nonword auditory stimuli (Appendices A and B). All word and nonword stimuli were monosyllabic and conformed to a consonant-vowel-consonant (CVC) format, such as “cat.” The nonwords were created by replacing the final consonants (codas) with codas from other real words (e.g., “bell” became “besh”).

Each stimulus was recorded by a male speaker of American English from Northeast Ohio. All sound clips were digitally manipulated to have an exact duration of 500 milliseconds (ms), using the ‘stretch and pitch’ effect in Adobe Audition (Adobe Systems, Inc.), which corrects for pitch changes associated with temporal manipulation of sound clips.

#### Procedure

Participants were tested individually in the Language Research Laboratory. First, participants gave their signed consent and completed a participant information form. Next, participants responded using MouseTracker (Freeman and Ambady, 2010) to a block of baseline trials and to a block of lexical decision trials. Participants were then given a distractor task (math test), followed by a second – target – block of lexical decision trials (including primed and unprimed words), and another block of baseline trials. In the baseline trials (12 in each block), participants were simply instructed to click on one of two response alternatives labeled “Here.” In the lexical decision task, participants were instructed to indicate whether the sound presented on each trial was a real English word or a nonword by clicking one of two appropriately labeled response options as quickly and as accurately as possible. This lexical decision task is considered to be relatively difficult, given that the nonwords are quite similar to the real words (only the coda differs).

At the beginning of each trial “START” appeared at the bottom-center of the screen, and the response options appeared in the top left and right corners of the screen. Clicking “START” cued the onset of the auditory stimulus and the response timer. Participants then clicked one of two buttons at the top right and left corners of the screen labeled “Word” and “Nonword,” respectively. If a participant took longer than 500  ms to initiate moving the mouse, a warning appeared at the end of that trial instructing the participant to start moving the mouse earlier on future trials. Participants completed four practice trials (two words and two nonwords) to become familiarized with the task. The prime block consisted of 80 trials, with 40 words and 40 nonwords. After completing the prime block, participants were asked to work on a math test for 3–5 min, which was simply included as a filler task. Following the math test, participants completed the target block, which consisted of 160 trials. Of the 160 trials, 80 trials were primed condition trials (40 words and 40 nonwords), where the same auditory stimulus was heard in both the prime and target blocks, and 80 trials were unprimed condition trials (40 new words and 40 new nonwords, i.e., stimuli that had not been presented in the prime block). Across participants, the stimuli were counterbalanced across two versions of the experiment. Specifically, words 1–40 were primed and words 41–80 were unprimed for half of the participants, and words 1–40 were unprimed and words 41–80 were primed for the other half of the participants. All stimuli, both words and nonwords, were randomly presented during the prime and target blocks of the experiment. Finally, participants were debriefed. The study took approximately 30 min to complete.

#### Design

All confirmatory statistical analyses were limited to responses to words in the target block. The effects of the manipulated within-participants’ variable “Priming” (primed/unprimed) were evaluated on three dependent variables: accuracy, reaction time, and mouse movement trajectories. Accuracy is the number of trials correctly receiving a “Word” response. Only correct responses were included in the analyses for all the other measures. Reaction time was measured from the moment the participant clicked on the “START” button until the participant clicked on the final “Word” response. Mouse movement trajectories were examined by comparing the *x*-coordinates over time for the primed word condition and the unprimed word condition.

### Results

We used mixed-effects models (Baayen et al., 2008) in R version 3.5.1. (R Core Team, 2018) to analyze the data, using the lme4 library (version 1.1-18-1; Bates et al., 2015). We used glmer to evaluate whether Priming had an effect on number of correct responses, and lmer to evaluate whether Priming had an effect on reaction times, or the overall mouse trajectory. We included Participants and Words as random effects. Furthermore, we added Priming as a random effect by Participants. For the analyses of the mouse trajectory (*x*-coordinates over time), we looked at the first 2,000 ms, and we centered “Time” and included it as a random and fixed effect (see Mirman, 2014). Mixed effects analyses were performed with Participants and Words crossed at the same level of sampling. An effect was interpreted when it improved model fit (the chi-square for the model had a *p* < 0.05) and the estimate with the standard error was reported for each effect. All analyses were aimed at investigating whether participants responded to primed words more efficiently than to unprimed words. Mean and standard error reaction times were extracted using emmeans (Lenth et al., 2022). See Table 1 for a summary of the descriptive statistics for the primed and unprimed conditions for words and nonwords.

**Table 1:** Mean reaction times (in ms) and percent errors (PE) for words and nonwords by priming for Experiment 1.

	Words		Nonwords	
	M (SE)	PE	M (SE)	PE
Primed	1,208.47 (23.10)	11.36%	1,309.72 (21.35)	13.48%
Unprimed	1,270.46 (22.40)	14.51%	1,307.90 (20.00)	10.38%

#### Preregistered confirmatory analyses

##### Accuracy

We predicted greater accuracy (fewer incorrect responses) in the primed word condition than in the unprimed word condition. Model comparisons indicated that priming significantly improved model fit, χ^2^_(1)_ = 12.49, *p*  < 0.001. As predicted, there were fewer errors in the primed (11.36% – 209 errors out of 1840 trials) than in the unprimed (14.51% – 267 errors out of 1840 trials) condition.

##### Reaction Times

We predicted faster reaction times in the primed word condition than in the unprimed word condition. Model comparisons indicated that priming significantly improved model fit, χ^2^_(1)_= 19.81, *p* < 0.001. Participants responded 62 ms (*Estimate*  =  61.99, *SE*  = 13.69) faster to words in the primed condition than to words in the unprimed condition.

##### Mouse Movement Trajectories

**Figure 1:**
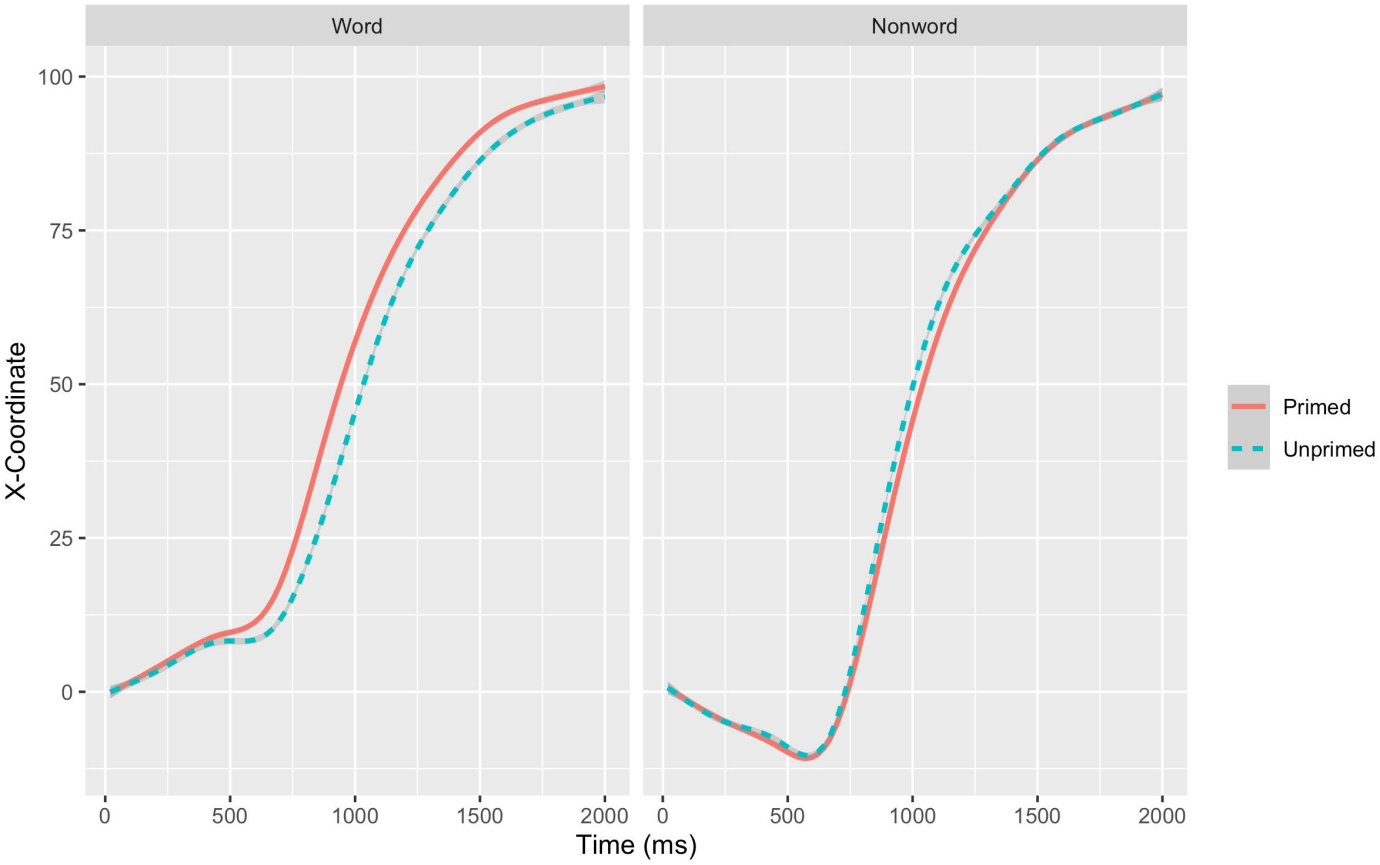
The initial 2,000  ms of the mouse trajectories for primed and unprimed words and nonwords in Experiment 1.

We predicted differences in the intercept, slope, or both, such that the primed word condition would be more efficient (higher intercept, steeper slope, or both) than the unprimed word condition. Model comparisons indicated that there was a significant effect of priming on the intercept, χ^2^_(1)_  =  8.53, *p* = 0.003, but not on the slope, χ^2^_(1)_  =  01, *p*  =  0.907, as priming did not interact with time. That is, priming significantly influenced the intercept but not the slope of the mouse trajectory. Overall, participants’ trajectories to words in the primed condition were more efficient (*Estimate* = −3.56, *SE*  = 1.67) than participants’ trajectories to words in the unprimed condition. Figure 1 depicts the unfolding of the first 2,000 ms of the mouse trajectories. As illustrated in Figure 1, for words (left panel), responses in the primed condition (continuous line) moved earlier toward the correct response (+100 *x*-coordinate) than responses in the unprimed condition (discontinuous line).

Our preregistration also included an alternative possibility regarding the mouse trajectories. The alternative possibility is that the mean trajectory for the primed condition crosses the mean trajectory for the unprimed word condition. For example, the advantage for the primed word condition could emerge later in the trajectory, with the earlier portion of the trajectory demonstrating no such advantage, and possibly even a disadvantage (reflecting earlier processes devoted to retrieving the memory, which leads to the eventual later repetition priming benefit). As it is possible to observe in Figure 1, the lines did not cross, so this alternative possibility was not supported by the data in Experiment 1.

#### Additional exploratory analyses

Previous researchers investigating the effects of long-term priming in words and nonwords with visual (e.g., Wagenmakers et al., 2004; Zeelenberg et al., 2004; Perea et al., 2016, 2020) and auditory (e.g., Sumner and Samuel, 2007) lexical decision tasks have found inconsistent results regarding long-term priming for nonwords, with reports of both facilitatory (e.g., Wagenmakers et al., 2004; Zeelenberg et al., 2004; Perea et al., 2016, 2020) and inhibitory (e.g., Wagenmakers et al., 2004; Zeelenberg et al., 2004; Sumner and Samuel, 2007; Perea et al., 2016, 2020) effects. Although Experiment 1 was designed with a focus on words, we included lexicality into our analyses on accuracy, reaction times, and mouse movement trajectories to further explore the effect of priming in nonwords. When a significant higher order lexical interaction was obtained, we analyzed the nonwords separately.

##### Accuracy

Model comparisons indicated that neither lexicality, χ^2^_(1)_ =  0.20, *p* =  0.652, nor priming, χ^2^_(1)_ =  0.02, *p* =  0.883, significantly improved model fit, but the Lexicality × Priming interaction significantly improved model fit, χ^2^_(1)_ =  20.22, *p* < 0.001. For nonwords, model comparisons indicated that priming significantly improved model fit, χ^2^_(1)_ =  16.04, *p* < 0.001. Specifically, there were more errors in the primed (13.48% - 248 errors out of 1840 trials) than in the unprimed (10.38% - 191 errors out of 1840 trials) condition for the nonwords.

##### Reaction Times

Model comparisons indicated that lexicality, χ^2^_(1)_ =  31.52, *p* < 0.001, priming, χ^2^_(1)_ =  9.88, *p* =  02, and the Lexicality  ×  Priming interaction, χ^2^_(1)_ =  11.43, *p* <  0.001, all significantly improved model fit. For nonwords, model comparisons indicated that priming did not significantly improve model fit, χ^2^_(1)_ =  0.02, *p* =  0.885.

##### Mouse Movement Trajectories

Model comparisons indicated that lexicality, χ^2^_(1)_ =  93.27, *p* < 0.001, the Time × Lexicality interaction, χ^2^_(1)_ =  1,941.64, *p* < 0.001, and the Time × Lexicality × Priming interaction, χ^2^_(3)_ =  549.07, *p* < 0.001, all significantly improved model fit, but priming, χ^2^_(1)_ =  2.44, *p* =  0.118, did not significantly improve model fit. For nonwords, model comparisons indicated that neither the priming, χ^2^_(1)_ =  0.51, *p* =  0.477, nor the Time × Priming interaction, χ^2^_(1)_ =  0.03, *p* =  0.864, significantly improved model fit.

### Discussion

We used computer mouse tracking to examine long-term repetition priming effects in an auditory lexical decision task. Across all of the dependent variables investigated – accuracy, reaction time, and mouse trajectory – participants responded more efficiently to primed than to unprimed words, consistent with the preregistered hypotheses. One additional observation worth noting is that the priming effect was observed on the intercept, as opposed to interacting with Time (i.e., there was no effect on the slope). Had the effect of priming changed over the course of the trial, such that the priming effect became larger or smaller throughout the trial, a significant interaction with Time would have been obtained.

As is clear from the inspection of Figure 1, the finding that words were responded to more efficiently in the primed condition compared to the unprimed condition did not emerge in nonwords, which is inconsistent with previous research demonstrating that repetition of nonwords can lead to greater familiarity (e.g., see Zeelenberg et al., 2004). However, primed nonwords had more errors than unprimed nonwords, which is consistent with the inhibitory effects found in Wagenmakers et al. (2004), Zeelenberg et al. (2004), and Perea et al. (2020). A design choice merits discussion. We decided to have the “Word” response always appear in the top-right corner of the screen, and the “Nonword” response always appear in the top left-corner of the screen. Doing so allowed for a straightforward comparison between primed and unprimed words, which was the primary purpose of the current study; however, this design also limits comparisons between words and nonwords. In Experiment 2, we counterbalanced the position of the response options, such that half of the participants responded to “Nonword ----- Word” and half the participants responded to “Word ----- Nonword.”

## Experiment 2

This experiment is similar to Experiment 1, except we counterbalanced the word/nonword response options, and we collected the data online instead of in person.

### Method

#### Participants

A total of 184 undergraduate participants^2^ (126 women, one genderfluid, two non-binary, *M*_Age_ = 20.30, *SE*_Age_ = 0.33) from Cleveland State University were recruited in exchange for research participation credit. All participants were right-handed, native speakers of American English, with no reported hearing or speech disorders. Additionally, we only analyzed data from participants who reported being at least 18 years old and agreed at the end of the study that we could use their data. Since data were collected online, participants could have started the experiment multiple times before completing the experiment. If participants started the priming block and did not complete the experiment, their data (including from any subsequent attempts) were excluded. We analyzed data from the first 46 participants in each of the four versions of the experiment that met these requirements. All participants provided their digital informed consent before participation and the procedures were approved by the Institutional Review Board at Cleveland State University.

#### Stimuli

We used the same stimuli as Experiment 1.

#### Procedure

Experiment 2 was implemented online using Labvanced (Finger et al., 2017). Across participants, the stimuli and the positions of the labels “WORD” and “NONWORD” were counterbalanced across four versions of the experiment. Also, participants were not warned to start moving sooner if they took longer than 500 ms to initiate movement. Before participants were debriefed, participants completed a post-experiment questionnaire. The post-experiment questionnaire included questions about the purpose of the experiment, whether participants had trouble hearing or understanding the stimuli, whether they thought we should use their data, etc. Other than these differences, the procedure was the same as Experiment 1.

#### Design

The design was the same as Experiment 1.

### Results

We used mixed-effects models (Baayen et al., 2008) in R version 4.1.2. (R Core Team, 2021) to analyze the data, using the lme4 library (version 1.1–28; Bates et al., 2015). We used glmer to evaluate whether Priming had an effect on number of correct responses, and lmer to evaluate whether Priming had an effect on reaction times, or the overall mouse trajectory.^3^ We included Participants and Words as random effects. Furthermore, we added Priming as a random effect by Participants. For the analyses of the mouse trajectory (*x*-coordinates over time), we analyzed the first 1,500 ms, — which differed from Experiment 1 because of differences in how the data were recorded in Labvanced (Finger et al., 2017) — and we centered “Time” and included it as a random and fixed effect (see Mirman, 2014). Mixed effects analyses were performed with Participants and Words crossed at the same level of sampling. An effect was interpreted when it improved model fit (the chi-square for the model had a *p* < 0.05). All analyses were aimed at investigating whether participants responded to primed words more efficiently than to unprimed words. Mean and standard error reaction times were extracted using emmeans (Lenth et al., 2022). See Table 2 for a summary of the descriptive statistics for the primed and unprimed conditions for words and nonwords.

**Table 2:** Mean reaction times (in ms) and percent errors (PE) for words and nonwords by priming for Experiment 2.

	Words		Nonwords	
	M (SE)	PE	M (SE)	PE
Primed	1,465.10 (27.14)	10.39%	1,605.25 (28.58)	14.09%
Unprimed	1,534.15 (29.00)	12.65%	1,589.63 (26.98)	12.95%

#### Preregistered confirmatory analyses

##### Accuracy

We predicted greater accuracy (fewer incorrect responses) in the primed word condition than in the unprimed word condition. Model comparisons indicated that priming significantly improved model fit, χ^2^_(1)_ = 20.97, *p* < 0.001. As predicted, there were fewer errors in the primed (10.39% - 765 errors out of 7,360 trials) than in the unprimed (12.65% - 931 errors out of 7,360 trials) condition.

##### Reaction Times

We predicted faster reaction times in the primed word condition than in the unprimed word condition. Model comparisons indicated that priming significantly improved model fit, χ^2^_(1)_ = 22.75, *p* < 0.001. Participants responded 69 ms (*Estimate* = 69.04, *SE* = 14.46, *t* = 4.78) faster to words in the primed condition than to words in the unprimed condition.

##### Mouse Movement Trajectories

**Figure 2:**
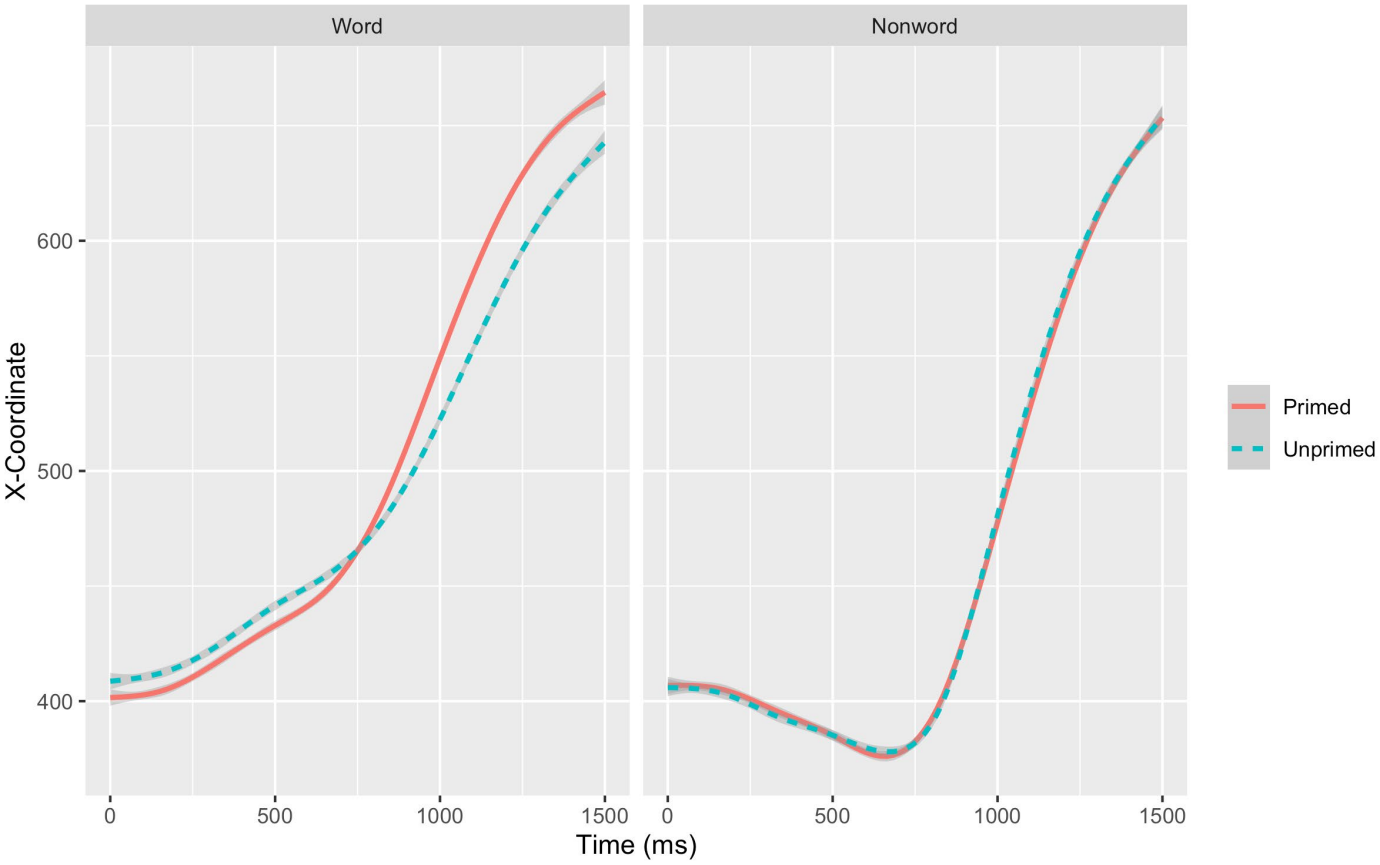
The initial 1,500  ms in Experiment 2 of the mouse trajectories for primed and unprimed words and nonwords.

We predicted differences in intercept, slope, or both, such that the primed word condition would be more efficient (higher intercept, steeper slope, or both) than the unprimed word condition. Model comparisons indicated that there was no significant effect of priming on the trajectory, χ^2^_(1)_ < 0.01, *p* = 0.964, but that priming did significantly interact with time, χ^2^_(1)_ = 8.35, *p* = 0.004. That is, priming did not significantly influence the intercept but did significantly influence the slope of the mouse trajectory. Overall, participants’ trajectories to words in the primed condition had a steeper slope (*Estimate* = −12.51, *SE* = 4.30, *t* = −2.91) than participants’ trajectories to words in the unprimed condition. Figure 2 depicts the unfolding of the first 1,500 ms of the mouse trajectories. As illustrated in Figure 2, for words (left panel), responses in the primed condition (continuous line) moved earlier toward the correct response (+800 *x*-coordinate) than responses in the unprimed condition (discontinuous line).

Our preregistration also included an alternative possibility regarding the mouse trajectories. The alternative possibility is that the mean trajectory for the primed condition crosses the mean trajectory for the unprimed word condition. For example, the advantage for the primed word could emerge later in the trajectory, with the earlier portion of the trajectory demonstrating no such advantage, and possibly even a disadvantage (reflecting earlier processes devoted to retrieving the memory, which leads to the eventual, later repetition priming benefit). As it is possible to observe in Figure 2, there is a trend that follows this pattern of results: the lines cross at about 750 ms after stimulus onset, when the primed overcomes the unprimed condition. In the initial section of the trajectory, the primed condition seems to be at a disadvantage, in line with the possibility that retrieving the memory exerts an initial delay that is compensated for at later stages in the trajectory. While this alternative possibility was not supported by the data in Experiment 1, this trend emerges in Experiment 2.

#### Additional exploratory analyses

Following Experiment 1’s additional exploratory analyses, we included lexicality in our analyses on accuracy, reaction times, and mouse movement trajectories to further explore the effect of priming in nonwords.^4^ When there was a significant higher order lexical interaction, we analyzed the nonwords separately. Three reaction times were removed because they were extreme outliers.^5^

##### Accuracy

Model comparisons indicated that lexicality, χ^2^_(1)_ =  6.89, *p* =  0.009, and the Lexicality × Priming interaction, χ^2^_(1)_ =  25.90, *p* <  0.001, significantly improved model fit, but priming did not significantly improve model fit, χ^2^_(1)_ =  0.66, *p* =  0.417. For nonwords, model comparisons indicated that priming significantly improved model fit, χ^2^_(1)_ =  18.14, *p* < 0.001. Specifically, for nonwords, there were more errors in the primed (14.09% - 1,037 errors out of 7,360 errors) than in the unprimed (12.95% - 953 errors out of 7,360 trials) condition.

##### Reaction Times

Model comparisons indicated that lexicality, χ^2^_(1)_ =  50.63, *p* < 0.001, priming, χ^2^_(1)_ =  9.65, *p* =  0.002, and the Lexicality × Priming interaction, χ^2^_(1)_ =  22.20, *p* < 0.001, all significantly improved model fit. For nonwords, model comparisons indicated that priming did not significantly improve model fit, χ^2^_(1)_ = 2.24, *p* = 0.135.

##### Mouse Movement Trajectories

Model comparisons indicated that lexicality, χ^2^_(1)_ =  148.65, *p* < 0.001, the Time × Lexicality interaction, χ^2^_(1)_ =  341.25, *p* < 0.001, and the Time × Lexicality × Priming interaction, χ^2^_(3)_ =  486.84, *p* < 0.001, all significantly improved model fit, but priming, χ^2^_(1)_ =  0.09, *p* =  0.767, did not significantly improve model fit. For nonwords, model comparisons indicated that neither priming, χ^2^_(1)_ =  0.01, *p* =  0.923, nor the Time × Priming interaction, χ^2^_(1)_ < 0.01, *p* =  0.962, significantly improved model fit.

### Discussion

We used computer mouse tracking to examine long-term repetition priming effects in an online auditory lexical decision task. Across all of the dependent variables investigated – accuracy, reaction time, and mouse trajectories – participants responded more efficiently to primed than to unprimed words, consistent with the results found in Experiment 1. We found that reaction times were longer in Experiment 2, consistent with other studies that have compared in person and online experiments (see, e.g., Fairs and Strijkers, 2021; Dufour et al., 2022), which could be due to a variety of differences between the two testing environments, such as the possibility of more environmental distractions, greater variability in participants’ computers, and a more heterogenous group of participants online compared to in person experiments. Two additional observations are worth noting. First, as illustrated in Figure 2, the priming effect was observed on the slope (i.e., the priming effect became larger over the course of the trial) whereas the priming effect was observed on the intercept in Experiment 1. Second, as illustrated in Figure 2, long-term repetition priming effects in the current mouse tracking study appear to emerge at approximately 875  ms, which is 375  ms longer than what was found in Experiment 1. This difference could be due to the fact that in Experiment 2, participants were not warned to start moving earlier if they took longer than 500  ms to initiate movement. Future studies should further investigate what factors influence the timing at which these effects emerge.

Recall that we counterbalanced the position of the response options, such that half of the participants respond “Nonword ----- Word” and half the participants respond to “Word ----- Nonword,” which would have allowed for an exploratory analysis directly comparing the priming effect between words and nonwords. Nevertheless, as is evident in Figure 2, once again replicating the pattern obtained in Experiment 1, the priming effect only emerged in the words, which is inconsistent with Zeelenberg et al. (2004) finding that repetition of nonwords can lead to greater familiarity. However, once again primed nonwords had more errors than unprimed nonwords, which is consistent with the inhibitory effects found in Wagenmakers et al. (2004), Zeelenberg et al. (2004), and Perea et al. (2020).

## General discussion

The mouse tracking data reported here open the door to new investigations aimed at understanding how the advantage for primed items relative to unprimed items unfolds over time. More specifically, we are able to observe the trajectories over time, which shed light on the decision processes, including gaining a better understanding of effects that emerge relatively earlier and later during processing. One cognitive mechanism underlying the repetition effect is the decision point, a post-lexical access process, which we can observe using mouse tracking. Although it may be premature to make comparisons between mouse tracking, the EEG technique, and eye-tracking (another behavioral measure), as well as other inferences about stages of processing (e.g., pre-lexical vs. lexical), the current study lays the foundation for such comparisons in future work. Knowing that long-term repetition priming effects are detectable in a number of mouse tracking dependent variables, including mouse trajectories, researchers can build on the design used in the current investigation to ask new questions of theoretical interest. For example, different aspects of mouse tracking data (e.g., initiation times, trajectories) might help to distinguish between competing accounts of long-term priming. We agree with previous accounts (e.g., MᶜLennan and Luce, 2005) that the repetition priming effect arises because of repeated activation of form-based representations in memory, and that the resonances in an adaptive resonance framework (see, e.g., Grossberg and Stone, 1986) may be the locus of facilitative priming effects. Although how mouse-tracking data might correspond to the adaptive resonance framework is beyond the scope of this paper, the current study sets the stage for future work connecting empirical mouse-tracking data with various theoretical accounts.

It is also important to highlight that we found the same pattern of results for Experiment 1, which was collected in person, and Experiment 2, which was collected online. More specifically, across both experiments we found that long-term repetition priming effects for words were facilitatory (i.e., more efficient for primed than unprimed words) and that long-term repetition priming effects for nonwords were inhibitory (i.e., more errors for primed than unprimed nonwords). When observing mouse-tracking trajectories it is possible to observe the lines crossing, which opens up new possibilities to explore the timing of word recognition. The “crossing” of the trajectories only emerged in Experiment 2 (not in Experiment 1) so any conclusions are preliminary and need to be taken under consideration cautiously. Nevertheless, if this trend were replicated in future studies, it opens up the possibility that the same condition (e.g., priming) can be detrimental at some point in the trajectory (e.g., earlier when retrieval is taking place) and facilitatory at some other point in the trajectory (e.g., later when priming is facilitating processing). This type of pattern has been reported elsewhere (Incera and MᶜLennan, 2016). These nuanced effects would be impossible to measure with an overall measure like accuracy or response time. Thus, continuous measures, such as mouse tracking, are necessary to uncover the timing of these cognitive processes.

The current study, which was powered for analyses comparing primed and unprimed words and included exploratory analyses with lexicality as a factor and examining priming effects in nonwords, may inform future studies that are appropriately powered for investigations with lexicality as part of the experimental design that include direct comparisons of priming effects between words and nonwords. For nonwords, the previously reported differences between facilitatory and inhibitory effects may be explained by task, design, task instructions, or some combination. For example, Perea et al. (2020) found inhibitory repetition effects for nonwords regardless of the instructions when the block was long (e.g., more stimuli presented), which is consistent with the results we obtained in the current study.

In addition to specific questions of interest to researchers investigating spoken word recognition, such as the time course of talker effects, the current results have implications for a variety of disciplines, including psychological science, linguistics, speech and hearing, and other related areas, in which priming has been used as a tool to investigate effects of theoretical interest. Just as the long-term repetition priming paradigm has been used to address a variety of empirical and theoretical questions (memory, language, clinical implications, etc.), the results of the current study – and the notion that mouse tracking can be effectively used when combined with the long-term repetition priming paradigm – should be applicable to a wide variety of research questions.

## Data availability statement

The data and the scripts to reproduce the analyses for Experiment 1 and Experiment 2 and the research materials for Experiment 1 are available at the Open Science Framework: https://osf.io/urtae/?view_only=66571c0c4e074cfc8071e7ccedbd3a19. Experiment 2 research materials and design are available at Labvanced: https://www.labvanced.com/page/library/43008.

## Ethics statement

The studies involving human participants were reviewed and approved by the Institutional Review Board at Cleveland State University. The participants provided their written/digital informed consent to participate in this study.

## Author contributions

ST, SI, and CM contributed to conception and design of the study. ST and SI performed the statistical analyses and wrote sections of the manuscript. CM wrote the first draft of the manuscript. All authors contributed to the article and approved the submitted version.

## Conflict of interest

The authors declare that the research was conducted in the absence of any commercial or financial relationships that could be construed as a potential conflict of interest.

## Publisher’s note

All claims expressed in this article are solely those of the authors and do not necessarily represent those of their affiliated organizations, or those of the publisher, the editors and the reviewers. Any product that may be evaluated in this article, or claim that may be made by its manufacturer, is not guaranteed or endorsed by the publisher.

## Footnotes

^1^We used G*Power (Faul et al., 2007) to estimate the appropriate sample size. To run a *t* test between two dependent means (matched pairs), with one tail (primed should be more efficient than unprimed), an alpha of 0.05, power of 0.95, and assuming a medium effect size of 0.50, we needed a total sample size of 45 participants. There were two versions of the experiment, so we recruited 46 participants to have an even number of participants in each version.

^2^Given that Experiment 2 was conducted completely online, which we expected would increase variability, we decided to collect data from four times the number of participants as in Experiment 1 (see Moore et al., 2021).

^3^We performed additional analyses controlling for screen height and screen width for the reaction times and mouse movement trajectories. Controlling for these variables did not change the results.

^4^We performed additional analyses controlling for screen height and screen width for the reaction times and mouse movement trajectories. Controlling for these variables did not change the results.

^5^The patterns are the same with and without the extreme outliers (greater than 100,000  ms).
