# Supplementary material for: Examining long-term repetition priming effects in spoken word recognition using computer mouse tracking
Source: Front Psychol. 2023 Jan 5;13:1074784. doi: 10.3389/fpsyg.2022.1074784 (PMC9850077; doi:10.3389/fpsyg.2022.1074784)
Supplement: Supplementary file 1 [file Table_1.DOCX]

# Appendix A

**Real Word Stimuli**

| bile | hose | lurve | road |
| --- | --- | --- | --- |
| boat | hug | mead | seal |
| cheese | jar | meat | search |
| choice | jeep | mess | shun |
| chore | jerk | mile | sing |
| church | jet | moan | term |
| death | king | mope | theme |
| ditch | kiss | mouth | tip |
| dodge | kit | nag | town^[[1]](#footnote-1)^ |
| dog | knit | niche | veil |
| door | lace | none | wedge |
| fill | lack | pad | weed |
| fin | lag | pave | weep |
| foal | lamb | pep | wing |
| foam | leech | perk | wipe |
| foil | leek | pill | yacht |
| ham | liar | pit | yam |
| hat | load | poll | year |
| hood | loathe | pun | yearn |
| hop | luck | rip | youth |

1. *t^S* was a nonword that was mistakenly used as a word (*tush*) in Experiment 1 and was replaced with the word *town* in Experiment 2. [↑](#footnote-ref-1)
