# Supplementary material for: Examining long-term repetition priming effects in spoken word recognition using computer mouse tracking
Source: Front Psychol. 2023 Jan 5;13:1074784. doi: 10.3389/fpsyg.2022.1074784 (PMC9850077; doi:10.3389/fpsyg.2022.1074784)
Supplement: Supplementary file 2 [file Table_2.DOCX]

# Appendix B

**Nonword Stimuli in Klattese^[[1]](#footnote-1)^**

| bIJ | hev | p@b | Jig |
| --- | --- | --- | --- |
| bYp | h@J | pef | JRz |
| b^p | kIv | p@s | JEv |
| Cim | leb | poT | k@k |
| CRf | lRm | Tib | kib |
| dES | lEk | tIT | kEJ |
| dIJ | lib | veJ | kIb |
| dIT | lYg | vRf | kIf |
| dIv | loC | wiS | pof |
| dob | loJ | wiT | p^m |
| daC | mig | wEf | rYC |
| fId | mEp | wIb | rIz |
| fIp | mYf | wYJ | roS |
| fYz | moC | wIs | seC |
| foC | mWk | wIv | sif |
| fuf | mIb | yif | S^p |
| fYT | n@C | huG | sof |
| nog | neC | hav | scv |
| g^d | nuT | J@C | tig |
| heb | nIz | JeC | tRC |

1. Klattese is a computer-friendly system of phonetic transcription (see Vitevitch & Luce, 2004). [↑](#footnote-ref-1)
